# Early transplantation-related mortality after allogeneic hematopoietic cell transplantation in patients with acute leukemia

**DOI:** 10.1186/s12885-021-07897-3

**Published:** 2021-02-18

**Authors:** Seom Gim Kong, Seri Jeong, Sangjin Lee, Jee-Yeong Jeong, Da Jung Kim, Ho Sup Lee

**Affiliations:** 1 Department of Pediatrics, Kosin University College of Medicine, Busan, South Korea; 2 Department of Laboratory Medicine, Kangnam Sacred Heart Hospital, Hallym University College of Medicine, Seoul, South Korea; 3 Graduate School, Department of Statistics, Pusan National University, Busan, South Korea; 4 Department of Biochemistry, Kosin University College of Medicine, Busan, South Korea; 5 Institute for Medical Science, Kosin University College of Medicine, Busan, South Korea; 6 Department of Internal Medicine, Kosin University College of Medicine, 262 Gamcheon-ro, Seo-gu, Busan, South Korea

**Keywords:** Cumulative incidence rates, Transplantation-related mortality, Risk factor, Hematopoietic stem cell transplantation, Acute leukemia

## Abstract

**Background:**

Transplantation-related mortality (TRM) is a major obstacle in allogeneic hematopoietic cell transplantation (allo-HCT). Approximately 60–80% of TRM occurs early, within 100 days of transplantation.

**Methods:**

This was a nationwide population cohort study involving 5395 patients with acute leukemia who underwent allo-HCT between 2003 and 2015. Patient data were collected from the Korean National Health Insurance Service database. We investigated the cumulative incidence rates (CIRs) of early TRM at 50 and 100 days.

**Results:**

The CIRs of early TRM at 50 and 100 days were 2.9 and 8.3%, respectively. There was no decrease in the CIRs of early TRM over time. The early mortality was significantly higher in patients with more than 9 months between the diagnosis and transplantation (CIRs of TRM at 50, 100 days; 6.0, 13.2%), previous transplantations (CIRs of TRM at 50, 100 days; 9.4, 17.2%), and cord blood transplantation (CIRs of TRM at 50, 100 days; 6.1, 8.3%). The early TRM was significantly lower in patients who received iron chelation before transplantation (CIRs of TRM at 50, 100 days; 0.3, 1.8%).

**Conclusions:**

In conclusion, the overall CIR of early TRM was less than 10%. The predictable factors for early TRM included age, time from diagnosis to transplantation, the number of prior transplantations, the graft source, and previous iron chelation therapy.

**Supplementary Information:**

The online version contains supplementary material available at 10.1186/s12885-021-07897-3.

## Introduction

Allogeneic hematopoietic cell transplantation (allo-HCT) is one of the most important treatment strategies for high-risk patients with acute leukemia. Although the complications and mortality associated with transplantation have decreased in recent years, transplantation-related mortality (TRM) is still the major barrier to allo-HCT [1–3]. Many studies have found that 60–80% of TRM occurs within 100 days of transplantation [3–6]. Recent large-scale studies in North America and Europe reported that the TRM at 100 days significantly decreased after the year 2000 [1, 2]. In this context, according to the Center for International Blood and Marrow Transplant Research (CIBMTR) study data, the 100-day TRM of acute myeloid leukemia patients with in first complete remission transplanted with using myeloablative conditioning (MAC) regimens decreased from 15 to 6% in matched sibling donors, and from 37 to 14% in matched unrelated donors [1]. In the European Society for Blood and Marrow Transplantation (EBMT) study, the TRM at 100 days decreased from 21.1 to 13.6% [2].

Some common causes of early TRM included infection, toxicity, and graft-versus-host disease (GVHD). Even within the first 100 days after allo-HCT, the cause of death varies as a result of the timing. The authors of the EBMT study reported that early mortality should be divided into the first 30 days (very early), and 30–100 days (early) after transplantation [2]. Infections and other causes accounted for more than 80% of the deaths occurring within 30 days of transplantation. Disease recurrence and GVHD accounted for 15% of the deaths. In contrast, relapses and GVHD accounted for more than 50% of the deaths between 30 and 100 days. Among non-relapse mortality, the mortality from GVHD decreased over time. However, mortality from other causes, such as infection and organ toxicity, was not significantly reduced. Similarly, an Italian study reported that mortality from acute GVHD has decreased significantly since 2001 although the mortality from infection and multi-organ failure increased [7].

Therefore, the objective of this study was to investigate changes in early TRM in Korea, using a large dataset from the National Health Insurance Service (NHIS) and analyzing the cumulative incidence rates (CIRs) of early TRM at 50 and 100 days after allo-HCT in patients with acute leukemia. We also investigated acute leukemia and the causes of early mortality and the associated risk factors associated with early TRM.

## Methods

### Data collection

This study was a nationwide, population-level, historical cohort study of patients with acute leukemia who underwent allo-HCT. This data was obtained from the claims database of the NHIS of the Republic of Korea. South Korea has a universal health coverage system provided by the central government, which has been unified since 2000. The NHIS provides health insurance to more than 99% of the population. Accordingly, the NHIS has a comprehensive health database for diagnoses, treatments, procedures, and prescriptions. They provide these extensive data for use in research after the approval process. This study also obtained death related data, including the cause of death from Statistics Korea, which has a comprehensive database in connection with the NHIS. In South Korea, death registration is usually completed and confirmed by a physician. The institutional review boards of Kosin University Gospel Hospital approved this study and granted a waiver of informed consent from the study participants owing to the nature of the data from which private information was deleted. All methods were carried out in accordance with relevant guidelines and regulations along with the approval.

### Study populations

We selected patients diagnosed with acute leukemia who received allo-HCT from 2003 to 2015. This study includes data on both adults and pediatric patients. Transplant data registered with the NHIS were from patients who reached complete remission prior to transplantation. The recipients and donors were typed at the allelic level for *HLA-A, HLA-B, HLA-C*, and *HLA-DRB1* including those with fully matched or single-HLA locus mismatched transplants.

### Treatment and procedures

The conditioning intensity was defined as MAC when the total body irradiation (TBI) was administered for 4 days or longer, or when busulfan was administered for 3 days or longer. In contrast, the cases in which TBI was administered for less than 4 days or busulfan for less than 3 days were classified as reduced-intensity conditioning (RIC). Rabbit anti-thymocyte globulin (ATG; Sanofi-Aventis, Cambridge, MA) was administered to patients at various dosages to prevent GVHD. ATG was given in equally divided doses for 2 or 3 days from day − 3. All patients received calcineurin inhibitors, including cyclosporine or tacrolimus, with or without short-term methotrexate as immunosuppressants to prevent GVHD. Prophylaxis against infections included low-dose acyclovir, trimethoprim−sulfamethoxazole, antifungal agents (such as fluconazole), antibiotics (such as levofloxacin), and preemptive therapy with ganciclovir for patients with cytomegalovirus infection (on the basis of antigen or DNA testing). Half of the patients received ursodiol as prophylaxis against cholestasis.

### Statistical methods

The objectives of this study were to determine the CIRs of early TRM at 50 and 100 days after transplantation and to identify the causes of death and risk factors for early TRM. The CIR of early TRM was reported at a specific time after the transplant (day 50 and 100) in a landmark approach. Patients who underwent two or more transplants in that period were analyzed for the last transplant. The probabilities of mortality were estimated using cumulative incidence curves. We used the chi-square test for categorical data and independent *t*-test for continuous data. The causes of death were reported within 50 days of allo-HCT. We used maximally selected log-rank statistics in the maxstat function of the R software (version 3.3.2) to identify the optimal threshold to assess the survival outcomes for age and time from diagnosis to transplantation. We selected the optimal age and time from diagnosis cut-offs to be 40 years and 9 months, respectively. The probability of overall survival was estimated by the Kaplan−Meier method. Logistic regression was used for multivariate analysis. The statistical analysis was performed using the R statistical software (version 3.4.4; R Foundation for Statistical Computing) and SAS statistical analysis software (version 9.4; SAS Institute Inc., Cary, NC, USA). *P*-values < 0.05 with 2 sided test were considered statistically significant.

## Results

### Patient characteristics

The characteristics of 5395 patients in the two transplant periods (from 2003 to 2009 and from 2010 to 2015) are shown in Table 1. The mean age of all patients was 35.9 ± 16.6 years (range, 0–72 years) at the time of transplantation, and 55.1% were male. The mean age at the time of transplantation has also increased from 31.8 to 38.3 years. Since 2010, the period from diagnosis to transplantation has been longer than that in the past. The number of patients who have had two or more transplants has also increased. However, the number of patients who required a high number of red blood cell (RBC) and platelet transfusions before transplantation decreased. Iron chelation was performed in some patients whose ferritin level was 1000 ng/mL or higher due to red blood cells transfusion. The number of patients who received iron chelating agents before transplantation increased from 5.2 to 20.1%.

**Table 1:** Characteristics of patients who underwent allogeneic hematopoietic cell transplantation by time period (*N* = 5395)

Characteristics	Total (%)	2003–2009 (%)	2010–2015 (%)	*p*-value
Number	5395	1958	3437	
Recipient age, years				
Mean	35.9 ± 16.6	31.8 ± 16.2	38.3 ± 16.3	< 0.001
0–19	1145 (21.2)	532 (27.2)	613 (17.8)	< 0.001
20–39	1707 (31.7)	700 (35.7)	1007 (29.3)	
40–59	2229 (41.3)	679 (34.7)	1550 (45.1)	
≥ 60	314 (5.8)	47 (2.4)	267 (7.8)	
Recipient sex				
Male	2973 (55.1)	1074 (54.9)	1899 (55.3)	0.798
Female	2422 (44.9)	884 (45.1)	1538 (44.7)	
Diagnosis				
ALL	1905 (35.3)	673 (34.4)	1232 (35.8)	0.290
AML	3490 (64.7)	1285 (65.6)	2205 (64.2)	
Time from diagnosis to transplantation, months	8.8 ± 12.1	7.7 ± 8.2	9.5 ± 13.7	< 0.001
Previous transplantation (≥1)	331 (6.1)	87 (4.4)	244 (7.1)	< 0.001
RBC transfusion before HCT (≥3)	2324 (43.1)	889 (45.4)	1435 (41.8)	0.010
PLT transfusion before HCT (≥4)	2322 (43.0)	882 (45.0)	1440 (41.9)	0.027
Previous iron chelation therapy	793 (14.7)	102 (5.2)	691 (20.1)	< 0.001
Graft source				
Peripheral blood	4041 (74.9)	1093 (55.8)	2948 (85.8)	< 0.001
Bone marrow	1141 (21.1)	764 (39.0)	377 (11.0)	
Cord blood	213 (3.9)	101 (5.2)	112 (3.3)	
Conditioning intensity				
MAC	3453 (64.0)	1459 (74.5)	1994 (58.0)	< 0.001
RIC	1942 (36.0)	499 (25.5)	1443 (42.0)	
Conditioning regimen				
TBI-based	1754 (32.5)	629 (32.1)	1125 (32.7)	< 0.001
Busulfan-based	3272 (60.6)	1136 (58.0)	2136 (62.1)	
Non-TBI, Non-busulfan	369 (6.8)	193 (9.9)	176 (5.1)	
Use of ATG				
No	3061 (56.7)	1593 (81.4)	1468 (42.7)	< 0.001
Yes	2334 (43.3)	365 (18.6)	1969 (57.3)	

*ALL* Acute lymphocytic leukemia; *AML* Acute myeloid leukemia; *RBC* Red blood cell; *HCT* Hematopoietic cell transplantation; *PLT* Platelet; *MAC* Myeloablative conditioning; *RIC* Reduced-intensity conditioning; *TBI* Total body irradiation; *ATG* Antithymocyte globulin

Values are presented as means ± standard deviations or numbers of cases (%)

The graft source of HCT in Korea has been changed. The use of peripheral blood increased from 55.8 to 85.8% and bone marrow decreased from 39.0 to 11.0%. The use of MAC decreased from 74.5 to 58.0%, while the use of RIC increased from 25.5 to 42.0%. TBI and busulfan-based conditioning regimens were used in approximately one-third and two-thirds of the patients, respectively. The number of patients who used ATG increased from 18.6 to 57.3%.

### The overall CIRs of early TRM and causes of mortality

**Fig. 1:**
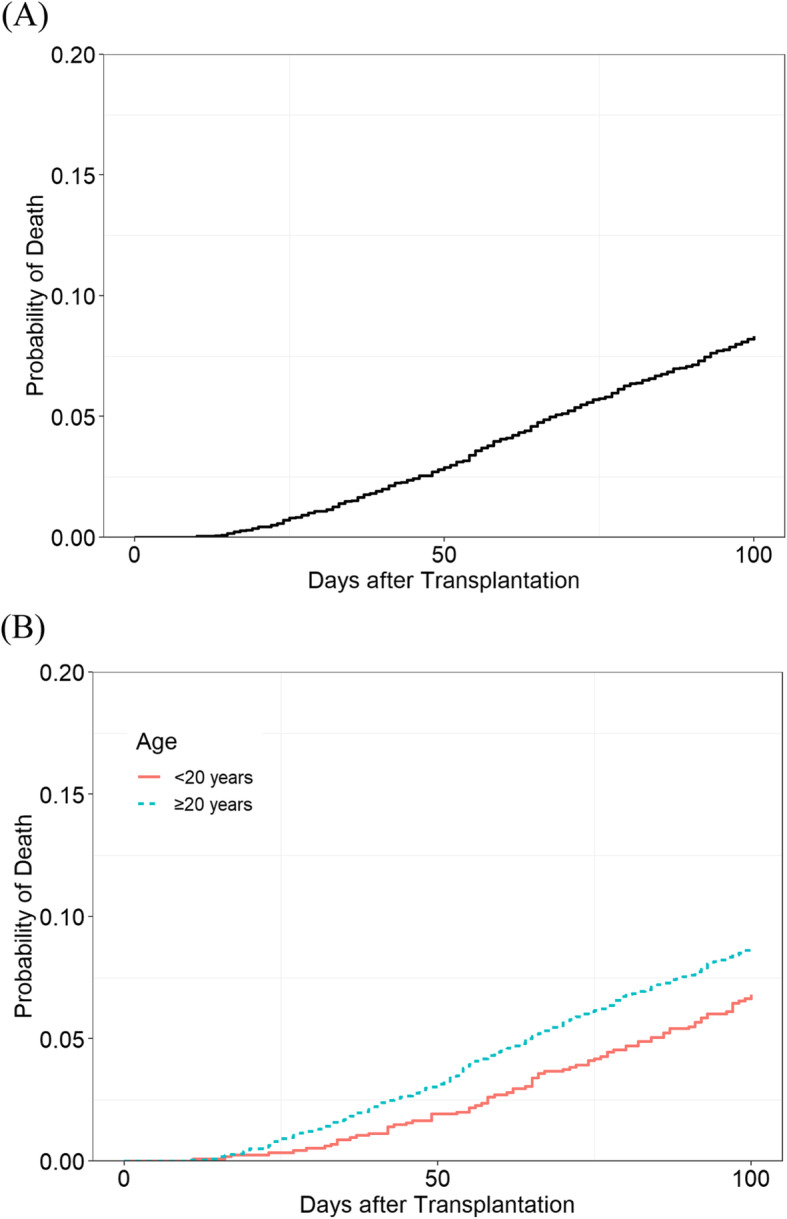
Cumulative incidence rates (CIRs) of early transplant-related mortality after HCT. **a** CIRs of early mortality at 50 and 100 days after transplantation in patients with acute leukemia were 2.9 and 8.3%, respectively. **b** In children and adults, the CIRs of early mortality at 50 days were 1.9 and 3.2% (*p* = 0.044), and the CIRs of early mortality at 100 days were 6.6 and 8.7% (*p* = 0.024), respectively

Accounting all, 151 and 442 patients died at 50 and 100 days after allo-HCT, respectively. The CIRs of early TRM were 2.9 and 8.3%, respectively (Fig. 1, Table 2). The CIRs of early mortality were significantly lower in those under 20 years of age. The median follow-up duration was 5.7 years (1–14.9). The 5-year overall survival (OS) rates were 54 ± 1% and 52 ± 1% in the transplantation periods of 2003–2009 and 2010–2015, respectively (*p* = 0.270). The 5-year OS rates for patient under 20 years of age were 56.7 ± 2.2% and 61.4 ± 2.6% in the transplantation periods of 2003–2009 and 2010–2015, respectively (*p* = 0.023, Supplemental Fig. 1). The 5-year OS rates for adults were 52.1 ± 1.3% and 49.0 ± 1.1% in the transplantation periods of 2003–2009 and 2010–2015, respectively (*p* = 0.104).

**Table 2:** The cumulative incidence rates of early transplant-related mortality after allogeneic hematopoietic cell transplantation

Variables	Total	Number of early TRM (%)			
		Within 50 days	*p*-value	Within 100 days	*p*-value
Total	5395	151 (2.9)		442 (8.3)	
Year of transplantation					
2003–2009	1958	47 (2.4)	0.210	160 (8.2)	1.000
2010–2015	3437	104 (3.0)		282 (8.2)	
Recipient age, years					
Mean	35.9 ± 16.6	39.1 ± 16.3	0.017	38.0 ± 16.9	0.005
0–19	1145	22 (1.9)	0.164	76 (6.6)	0.015
20–39	1707	47 (2.8)		127 (7.4)	
40–59	2229	73 (3.3)		213 (9.6)	
≥ 60	314	9 (2.9)		26 (8.3)	
Recipient sex					
Male	2973	69 (2.3)	0.023	239 (8.0)	0.685
Female	2422	82 (3.4)		203 (8.4)	
Diagnosis					
ALL	1905	65 (3.4)	0.054	179 (9.4)	0.020
AML	3490	86 (2.5)		263 (7.5)	
Time from diagnosis to transplantation, months					
< 9	4434	93 (2.1)	< 0.001	315 (7.1)	< 0.001
≥ 9	961	58 (6.0)		127 (13.2)	
Previous transplantations					
No	5064	120 (2.4)	< 0.001	385 (7.6)	< 0.001
Yes	331	31 (9.4)		57 (17.2)	
Previous iron chelation therapy					
No	4602	149 (3.2)	< 0.001	428 (9.3)	< 0.001
Yes	793	2 (0.3)		14 (1.8)	
Graft source					
Peripheral blood	4041	119 (2.9)	< 0.001	333 (8.2)	< 0.001
Bone marrow	1141	19 (1.7)		70 (6.1)	
Cord blood	213	13 (6.1)		39 (18.3)	
Use of ATG					
No	3061	77 (2.5)	0.173	229 (7.5)	0.033
Yes	2334	74 (3.2)		213 (9.1)	

*TRM* Transplant-related mortality; *ALL* Acute lymphocytic leukemia; *AML* Acute myeloid leukemia; *ATG* Antithymocyte globulin

Values are presented as means ± standard deviations or numbers of cases (%)

The common causes of early TRM within 50 days of allo-HCT are described in Supplemental Table 1. Infection-related death (66.9%) was the most common cause, such as pneumonia (43.7%) and sepsis (21.9%). Organ failure-related death (16.6%) was also common, including that due to kidney (7.3%), multi-organ (6.0%), and liver toxicity (2.6%). Other causes related to bleeding included intra-cranial hemorrhage (5.3%) and unknown causes (11.3%).

### Risk factors for early TRM

**Fig. 2:**
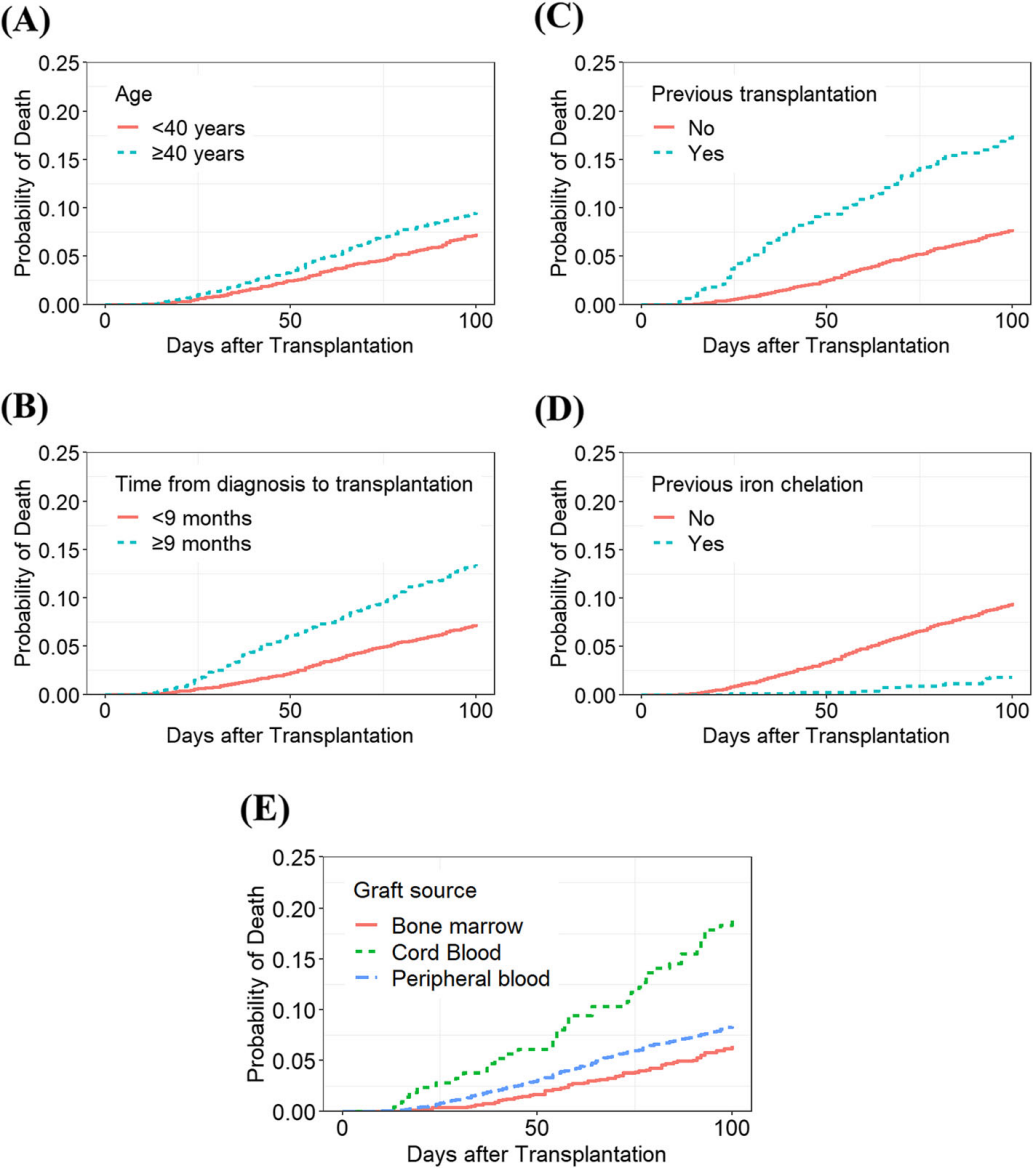
The comparison of cumulative incidence rates of early transplant-related mortality according to the variables. **a** age, (**b**) time from diagnosis to transplantation, (**c**) previous number of transplantations, (**d**) previous therapy with iron chelation, and (**e**) graft sources

The detailed results of the CIRs of early TRM at 50 and 100 days (according to other variables) are shown Table 2 and Fig. 2. Since 2010, the early mortality did not change between the two periods. There was no significant difference in early TRM within 50 days according to age. However, the TRM at 100 days was higher in those 40 years old or older (< 40 vs ≥40; 7.1 vs. 9.4%, *p* = 0.003). Early mortality was significantly higher in patients with more than 9 months between diagnosis and transplantation (CIRs of TRM at 50, 100 days; 6.0, 13%, respectively). In addition, patients who underwent one or more previous transplantations showed significantly higher CIRs of early TRM at 50 and 100 days (9.4, 17.2%, respectively).

The CIRs of early TRM were significantly lower in patients who received previous iron chelation therapy at 50, 100 days (0.3, 1.8%, respectively) compared to that of those who did not receive iron chelation therapy. The average number of RBC transfusions was higher in the iron chelation group (6.4 ± 8.9 vs 3.7 ± 5.4, data was not shown) than it was in other groups. The CIRs of early TRM were higher for patients whose graft source was cord blood at 50 and 100 days (6.1, 18.3%, respectively) than they were in patients with other graft sources.

The detailed results of univariate and multivariate analysis of early TRM at 50 and 100 days according to other variables are shown in Supplemental Table 2 and Table 3. The independent high-risk factors of early TRM included older age (≥40 years), longer duration from diagnosis to transplantation, previous transplantations, and cord blood transplantation. In particular, previous iron chelation therapy was an independent low-risk factor for early TRM (HR, 95% CI at 50 and 100 days; 0.07, 0.02–0.29, *p* < 0.001; 0.17, 0.10–0.29, *p* < 0.001).

**Table 3:** Multivariate analysis for early transplant-related mortality

Variables	Within 50 days			Within 100 days		
	HR	95% CI	*p*-value	HR	95% CI	*p*-value
Older age (≥40)	1.68	(1.20–2.36)	0.003	1.63	(1.34–1.98)	< 0.001
Diagnosis						
ALL	Reference			Reference		
AML	0.75	(0.54–1.05)	0.097	0.82	(0.68–1.00)	0.050
Longer D-to-HCT duration (≥9 months)	2.09	(1.39–3.13)	< 0.001	1.58	(1.23–2.03)	< 0.001
Previous transplantation	2.51	(1.55–4.08)	< 0.001	1.79	(1.28–2.49)	0.001
Previous iron chelation therapy	0.07	(0.02–0.29)	< 0.001	0.17	(0.10–0.29)	< 0.001
Graft source						
Peripheral blood	Reference			Reference		
Bone marrow	0.61	(0.37–1.01)	0.054	0.80	(0.61–1.04)	0.097
Cord blood	2.01	(1.11–3.64)	0.022	2.44	(1.73–3.45)	< 0.001
Use of ATG	1.07	(0.77–1.50)	0.690	1.17	(0.96–1.42)	0.125

*HR* Hazard ratio; *95% CI* 95% Confidence interval; *ALL* Acute lymphocytic leukemia; *AML* Acute myeloid leukemia; *D-to-HCT* Diagnosis to hematopoietic cell transplantation; *ATG* Antithymocyte globulin

## Discussion

Total hematopoietic cell transplantation conducted in South Korea doubled over 10 years from 1139 cases in 2005 to 2286 cases in 2015 [8]. During our study period, the frequency of allo-HCT in acute leukemia also increased. In addition, the age of patients, the use of peripheral blood, RIC regimen, and the experience of iron chelation significantly increased. Globally, with advances in supportive care and RIC regimen, transplantation in elderly and high-risk patients has also increased [1, 2, 7, 9, 10]. In North America and Europe, the average age of transplantation increased from 33 to 40 years since 2000 [1, 2]. According to the CIBMTR data from North America, the proportion of patients over 60 years old increased from 1% in 1994–1995 to 10% in 2004–2005 [1]. In addition, transplantations in patients with unrelated or mismatched donors, high risk disease status, and poor performance status increased [1, 7, 9, 11].

The CIRs of early TRM at 50 and 100 days for patients with acute leukemia between 2003 and 2015 were 2.9 and 8.3%, respectively. Other studies have reported the TRM at 100 days of 5–20%, and our results were similar [1, 2, 11]. In addition, many studies reported a significant decrease in the mortality of transplantation over time [1, 2, 7, 9, 10]. These studies explained these changes as a result of the use of less toxic conditioning, accurate HLA matching, advances in the prevention and treatment of GVHD, and improved engraftment with increased peripheral blood use [1, 7, 9, 10]. In our study, there was no significant decrease in early TRM over time. During this study period, the number of patients receiving iron chelation therapy increased, while the number of elderly patients and patients who had previous transplantation also increased. On the other hand, the use of bone marrow decreased. In addition, although we have not investigated, unrelated transplantation would have increased as in other studies [1, 9]. For this reason, we speculated that there was no significant improvement in early TRM in our study.

This study showed that the most common causes of death within 50 days of transplant were infection (pneumonia, sepsis) and organ failure. In previous studies, the most common causes of early TRM were infection (pneumonia), organ failure, GVHD, and relapse [2, 7]. However, similar to our findings, other groups have found that infection or organ failure were related to death at very early periods after transplantation [2, 12].

In this study, there were significantly higher CIRs of early TRM in the following settings: older age, a long time from diagnosis to transplantation, previous transplantations, the use of cord blood as a graft source, and the absence of iron chelation therapy before transplantation. Many studies have evaluated the risk factors related to TRM, including age, disease status, donor matching, stem cell source, and interval between transplants [3, 5–7, 9, 11–15]. Old age was an important risk factor for early TRM [1, 2, 6, 11]. In our study, age over 40 years old was a factor affecting early mortality. However, TRM was not higher in patients over 60 years old than it was in those under 60.

In addition, cord blood transplantation was associated with higher early TRM. Patients receiving cord blood usually had longer neutrophil recovery time than did those receiving bone marrow or peripheral blood [16]. The delayed engraftment can result in high early mortality caused by neutropenic fever or sepsis. Patients who underwent previous transplantations presented with relapse or refractory disease status after the first transplantation. Patients who underwent second transplantation showed non-relapse mortality greater than 30% [17, 18]. Patients with a high risk of relapse or refractory disease who underwent previous transplantations had a high incidence of TRM [12].

Another significant factor for TRM was iron chelation treatment. Iron overload was already known as a major adverse prognostic factor in transplantation of benign hematologic diseases such as thalassemia [19]. In addition, it has been reported to be associated with low survival and high TRM in allo-HCT of hematologic malignancies [20–25]. Pullarkat et al. found that early mortality at 100 days and the risk of death (due to acute GVHD and infection) increased when pre-transplantation ferritin levels were higher than 1000 ng/mL [23]. Deaths from iron overload were caused by organ toxicity and liver toxicity, such as venous occlusive disease [24, 26]. Iron chelation has been found to reduce mortality in patients who were at risk of high mortality due to iron overload [27, 28]. Sivgin et al. reported that peri-transplant mortality at 100 days after allo-HCT was 18.9% in patients who did not receive iron chelation and 2.3% in patients who received iron chelation therapy [27].

In our study, a total of 14.7% of patients received iron chelating agents before transplantation. The CIR of early TRM was significantly lower in patients who received iron chelating agents than it was in those who did not receive this therapy. We did not analyze the pre-transplant ferritin levels or the duration of iron chelation in this study. Although the average number of RBC transfusions was significantly higher in patients who underwent iron chelation than it was in those who did not, the mortality rate was low. Some authors have reported that severe iron overload itself was detrimental, but also that toxic non-transferrin-bound iron caused by conditioning was associated with tissue damage [20, 29]. Under this background, Armand et al. administered deferoxamine for 2 weeks before transplantation to 5 patients with median ferritin level of 3746 ng/mL [30]. Veno-occlusive disease did not occur in all of them and all survived until 22 months. In general, the iron overload rate of patients before receiving allo-HCT was as high as 30–70% [25, 31]. Although the use of iron chelation has increased, more active treatment for iron overload is needed.

This study had several limitations. First, it was limited to patients who were registered with the NHIS in Korea. In addition, because of the limitation of big data, we were not able to analyze disease status, donor type, recurrence, or detailed clinical findings. However, this study was a meaningful retrospective study that was based on large-scale transplant data conducted in Korea over 14 years. In conclusion, the CIRs of early TRM at 50 and 100 days were similar to those reported in previous studies (2.9 and 8.3%). The most common causes of death were infection and organ failure. The highest rates of early TRM were found in patients who were older, had a long period to transplantation, underwent previous transplantations, and received cord blood as the graft source. Patients who received iron chelation therapy before transplantation had a low incidence rate of early TRM.

## Supplementary Information

**Additional file 1.**

**Additional file 2.**

**Additional file 3.**

## Data Availability

The authors confirm that the data supporting the findings of this study are available within the article and its supplementary materials. Raw data generated at National Health Insurance Service are not available because of the personally identifiable information. If one researcher wants to access data, the researcher should submit the security memorandum and pledge to the Institutional Review Board of National Health Insurance Sharing Service. After approval, the researcher can receive data with blind identification and the data must be analyzed only in permitted rooms in centers of National Health Insurance Service. Contact information for a data access committee is listed as follows: National Health Insurance Sharing Service, Tel: 82–33–736-2432; Official internet site: https://nhiss.nhis.or.kr/bd/ay/bdaya001iv.do
