# Supplementary material for: Early transplantation-related mortality after allogeneic hematopoietic cell transplantation in patients with acute leukemia
Source: BMC Cancer. 2021 Feb 18;21:177. doi: 10.1186/s12885-021-07897-3 (PMC7891151; doi:10.1186/s12885-021-07897-3)

**Early transplantation-related mortality after allogeneic hematopoietic cell transplantation in patients with acute leukemia**

Seom Gim Kong^1^, Seri Jeong^2^, Sangjin Lee^3^, Jee-Yeong Jeong^4,5^, Da Jung Kim^6^, Ho Sup Lee^6^

^1^Department of Pediatrics, Kosin University College of Medicine, Busan, South Korea

^2^Department of Laboratory Medicine, Kangnam Sacred Heart Hospital, Hallym University College of Medicine, Seoul, South Korea

^3^Graduate School, Department of Statistics, Pusan National University, Busan, South Korea

^4^Department of Biochemistry, Kosin University College of Medicine, Busan, South Korea;

^5^Institute for Medical Science, Kosin University College of Medicine, Busan, South Korea;

^6^Department of Internal Medicine, Kosin University College of Medicine, Busan, South Korea

**Supplemental Fig. 1S.** The 5-year overall survival (OS) rates. (A) The 5-year OS rates for children (< 20 years old) were 56.7 ± 2.2% and 61.4 ± 2.6% in the transplantation periods of 2003–2009 and 2010–2015, respectively (*p* = 0.023). (B) The 5-year OS rates for adults (≥ 20 years old) were 52.1 ± 1.3% and 49.0 ± 1.1% in the transplantation periods of 2003–2009 and 2010–2015, respectively (*p* = 0.104).

(A)

**
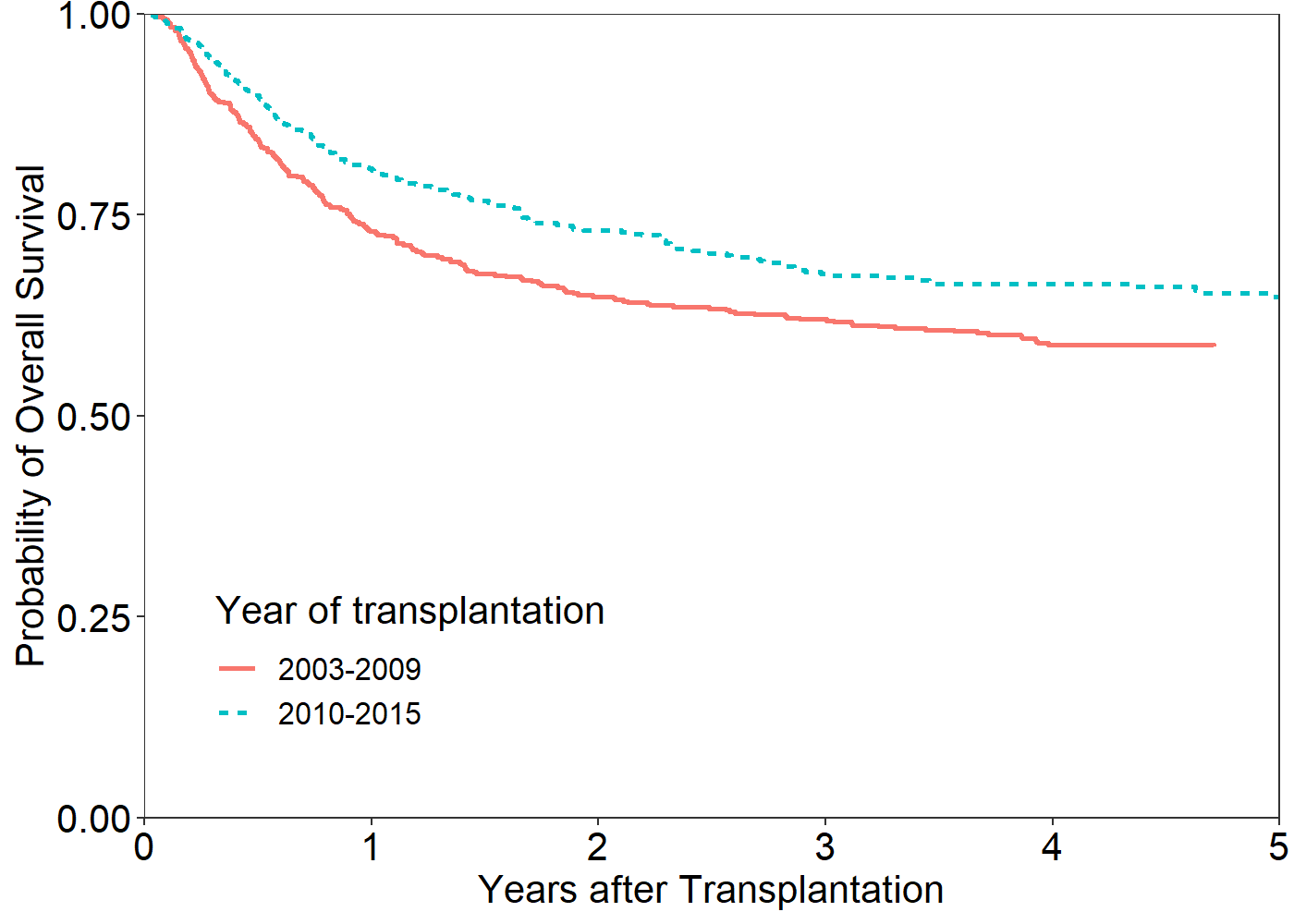
**

(B)


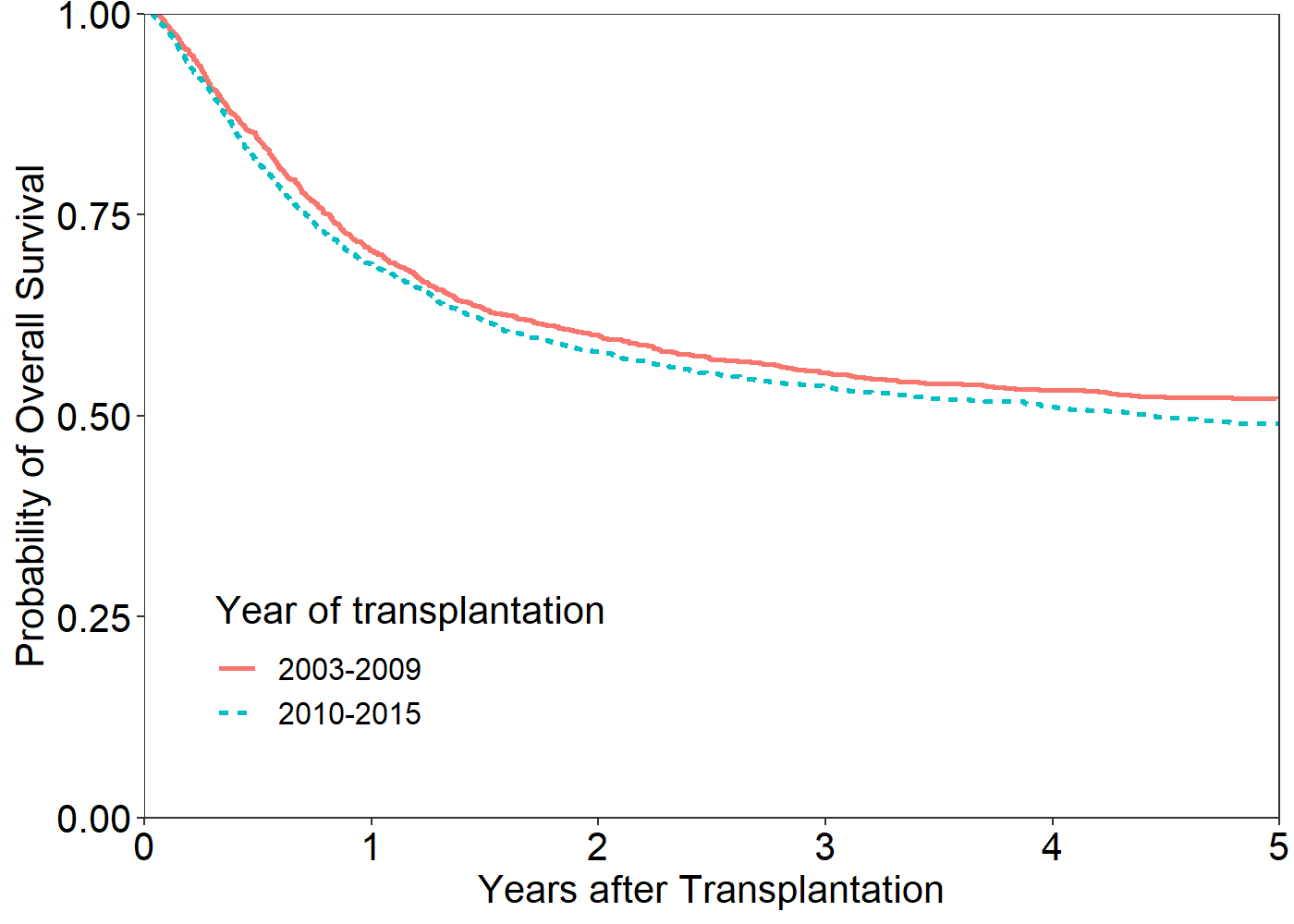

Supplement: Supplementary file 1 — Additional file 1. [file 12885_2021_7897_MOESM1_ESM.docx]
