# Supplementary material for: Early transplantation-related mortality after allogeneic hematopoietic cell transplantation in patients with acute leukemia
Source: BMC Cancer. 2021 Feb 18;21:177. doi: 10.1186/s12885-021-07897-3 (PMC7891151; doi:10.1186/s12885-021-07897-3)
Supplement: Supplementary file 2 — Additional file 2. [file 12885_2021_7897_MOESM2_ESM.docx]

**Early transplantation-related mortality after allogeneic hematopoietic cell transplantation in patients with acute leukemia**

Seom Gim Kong^1^, Seri Jeong^2^, Sangjin Lee^3^, Jee-Yeong Jeong^4,5^, Da Jung Kim^6^, Ho Sup Lee^6^

^1^Department of Pediatrics, Kosin University College of Medicine, Busan, South Korea

^2^Department of Laboratory Medicine, Kangnam Sacred Heart Hospital, Hallym University College of Medicine, Seoul, South Korea

^3^Graduate School, Department of Statistics, Pusan National University, Busan, South Korea

^4^Department of Biochemistry, Kosin University College of Medicine, Busan, South Korea;

^5^Institute for Medical Science, Kosin University College of Medicine, Busan, South Korea;

^6^Department of Internal Medicine, Kosin University College of Medicine, Busan, South Korea

**Supplemental Table 1. Main causes of death within 50 days of allogeneic hematopoietic cell transplantation (N=151)**

| Causes of death | Death within 50 days (%) |
| --- | --- |
| Infection related | 101 (66.9) |
| Pneumonia | 66 (43.7) |
| Sepsis | 33 (21.9) |
| Other infection | 2 (1.3) |
| Organ failure | 25 (16.6) |
| Cardiac | 1 (0.7) |
| Liver | 4 (2.6) |
| Kidney | 11 (7.3) |
| Multi-organ failure | 9 (6.0) |
| Others* | 8 (5.3) |
| Unknown | 17 (11.3) |

***Others; causes of death including bleeding and graft versus host disease, etc.**
