# Supplementary material for: Early transplantation-related mortality after allogeneic hematopoietic cell transplantation in patients with acute leukemia
Source: BMC Cancer. 2021 Feb 18;21:177. doi: 10.1186/s12885-021-07897-3 (PMC7891151; doi:10.1186/s12885-021-07897-3)
Supplement: Supplementary file 3 — Additional file 3. [file 12885_2021_7897_MOESM3_ESM.docx]

**Early transplantation-related mortality after allogeneic hematopoietic cell transplantation in patients with acute leukemia**

Seom Gim Kong^1^, Seri Jeong^2^, Sangjin Lee^3^, Jee-Yeong Jeong^4,5^, Da Jung Kim^6^, Ho Sup Lee^6^

^1^Department of Pediatrics, Kosin University College of Medicine, Busan, South Korea

^2^Department of Laboratory Medicine, Kangnam Sacred Heart Hospital, Hallym University College of Medicine, Seoul, South Korea

^3^Graduate School, Department of Statistics, Pusan National University, Busan, South Korea

^4^Department of Biochemistry, Kosin University College of Medicine, Busan, South Korea;

^5^Institute for Medical Science, Kosin University College of Medicine, Busan, South Korea;

^6^Department of Internal Medicine, Kosin University College of Medicine, Busan, South Korea

**Supplemental Table 2. Univariate analysis for early transplant-related mortality**

| Variables | Within 50 days | | | | Within 100 days | | |
| --- | --- | --- | --- | --- | --- | --- | --- |
|  | HR | 95% CI | *p-*value | HR | | 95% CI | *p-*value |
| Year of transplantation |  |  |  |  | |  |  |
| 2003-2009 | Reference |  |  | Reference | |  |  |
| 2010-2015 | 1.27 | (0.90-1.79) | 0.177 | 1.01 | | (0.83-1.23) | 0.913 |
| Older age (≥40) | 1.34 | (0.97-1.85) | 0.073 | 1.34 | | (1.11-1.62) | 0.002 |
| Female sex | 1.47 | (1.06-2.02) | 0.020 | 1.05 | | (0.87-1.27) | 0.607 |
| Diagnosis |  |  |  |  | |  |  |
| ALL | Reference |  |  | Reference | |  |  |
| AML | 0.72 | (0.52-0.99) | 0.045 | 0.79 | | (0.66-0.96) | 0.016 |
| Longer D-to-HCT duration (≥9 months) | 2.94 | (2.12-4.08) | <0.001 | 1.94 | | (1.58-2.38) | <0.001 |
| Previous transplantation | 4.14 | (2.79-6.14) | <0.001 | 2.44 | | (1.85-3.23) | <0.001 |
| Previous iron chelation therapy | 0.08 | (0.02-0.31) | <0.001 | 0.18 | | (0.11-0.31) | <0.001 |
| Graft source |  |  |  |  | |  |  |
| Peripheral blood | Reference |  |  | Reference | |  |  |
| Bone marrow | 0.56 | (0.35-0.91) | 0.019 | 0.73 | | (0.57-0.95) | 0.018 |
| Cord blood | 2.12 | (1.20-3.76) | 0.010 | 2.34 | | (1.68-3.27) | <0.001 |
| Use of ATG | 1.27 | (0.92-1.74) | 0.148 | 1.23 | | (1.02-1.48) | 0.029 |

HR, hazard ratio; 95% CI, 95% confidence interval; ALL, acute lymphocytic leukemia; AML, acute myeloid leukemia; D-to-HCT, diagnosis to hematopoietic cell transplantation; ATG, antithymocyte globulin.
